# “Our Home Is a Muddy Structure”: Perceptions of Housing and Health Risks Among Older Adults in Contrasting Neighborhoods in Ghana

**DOI:** 10.3389/fpubh.2021.650861

**Published:** 2021-04-22

**Authors:** Dominic A. Alaazi, Tania Stafinski, Joshua Evans, Stephen Hodgins, Martin Oteng-Ababio, Devidas Menon

**Affiliations:** ^1^School of Public Health, University of Alberta, Edmonton, AB, Canada; ^2^Earth and Atmospheric Sciences, Faculty of Science, University of Alberta, Edmonton, AB, Canada; ^3^Department of Geography and Resource Development, University of Ghana, Accra, Ghana

**Keywords:** aging, Ghana, health, neighborhoods, older adults, slums

## Abstract

Aging occurs in a variety of social and physical environmental settings that affect health. However, despite their rapidly growing populations, public health research in sub-Saharan Africa has yet to address the role of residential environments in the health and well-being of older adults. In this study, we utilized an ethnographic research methodology to explore barriers and facilitators to health among older adults residing in two contrasting neighborhoods in Accra, Ghana. Our specific objective was to identify patterns of health risks among older adults in the two neighborhoods. Data were collected through qualitative interviews with a purposive sample of health workers (*n* = 5), community leaders (*n* = 2), and older adults residing in a slum and non-slum neighborhood (*n* = 30). Our thematic data analysis revealed that, despite different underlying drivers, health barriers across the slum and non-slum were largely similar. The harmful effects of these health barriers – poor built environments, housing precariousness, unsanitary living conditions, defective public services, and social incivilities – were mitigated by several facilitators to health, including affordable housing and social supports in the slum and better housing and appealing doors in the non-slum. Our study contributes to a more nuanced understanding of the ways in which aging and urban environments intersect to influence population health in resource poor settings. In particular, rather than the commonly referenced dichotomy of poor and non-poor settlements in discourses of neighborhood health, our findings point to convergence of health vulnerabilities that are broadly linked to urban poverty and governmental neglect of the elderly.

## Introduction

The population of older adults is increasing globally; by 2050, the number of people aged 65 and older will reach 1.5 billion, up from 524 million in 2010 (1). The majority of this population will reside in urban areas of the developing world, where sharp increases in morbidity and mortality from non-communicable diseases have been recorded (2). In Ghana, recent gains in life expectancy underlie an unprecedented increase in the population of older adults, estimated to rise from 1.4 million in 2010 to ~6.3 million in 2050 (3). These demographic changes have catapulted aging issues onto national and global policy agendas, the majority of which now present programs and interventions to support aging-in-place at home and in neighborhood settings (4).

The focus on neighborhoods is important, given that older adults are less mobile and their duration of exposure to community environments is comparatively longer (5, 6). The health effects of adverse neighborhood conditions are therefore more salient for older populations than for younger demographics (7, 8).

The nature and quality of neighborhood built environments, including their spatial organization, land use patterns, and aesthetics, can be critical determinants of health among older adults. For example, poor location and visibility of road signage may accentuate cognition problems and discourage service utilization among older adults (7). Visible signs of environmental dereliction (e.g., dilapidated housing and disintegrated sidewalks) can intimidate older adults and cause them to refrain from outdoor activities (9).

Evidence from the United States suggests a positive relationship between the availability of pedestrian infrastructure and older adults' likelihood of walking (10). Neighborhoods with poor street connectivity and crumbling sidewalks can also restrict physical activity and contribute to disability and depressive symptoms in older adults (11).

Exposure to neighborhood social disorder, such as crime, excessive noise, graffiti, and street litter, has been found to be independently associated with reductions in physical activity, rising obesity prevalence, declines in physical functioning, and the onset of depressive symptoms among older adults. Fear of crime is an independent predictor for mobility declines (12), overweight (13), physical and functional disability (11, 14, 15), and depression in older adults (16). Among a sample of US older adults, Eisenstein and colleagues (13) found a strong association between fear of crime and risk of high BMI.

Not only can quantifiable physical and social features of neighborhoods affect health and well-being; so too can such symbolic constructs as place meaning, place identity, and sense of place. In their study examining sense of community, Zhang and Zhang (17) found a positive correlation between strong sense of community and subjective well-being among Chinese older adults. Similarly, Kitchen and colleagues (18) reported a strong association between sense of community belonging and mental health of older adults, after adjusting for geography and socioeconomic status.

While this body of literature has helped to advance public health knowledge of neighborhood effects on older adults' health, it is noticeably limited in geographical and methodological scope. First, with the exception of a few from Asia and Latin America [e.g., (19–21)], the literature examining relations between neighborhoods and older adults' health has largely neglected low and middle-income regions, such as sub-Saharan Africa (SSA), where much of the expected increase in older populations will occur. Second, this literature relies mostly on analyses of selected variables from large quantitative datasets that do not account for the full range of complex relationships between older adults and their neighborhood environments (16, 22). To build on the current literature and address these gaps, we utilized the person-environment (P-E) hypothesis and an ethnographic research methodology to explore health barriers and facilitators among a sample of older adults residing in two environmentally contrasting neighborhoods in Accra, Ghana. Our specific objective was to explain patterns of health risks among older populations in the city. The focus on urban neighborhoods is important and timely, given the rapid urbanization of older adults in the country (3). In the sections that follow, we discuss the P-E hypothesis and the methods used before presenting and discussing our findings.

## The Person-Environment Hypothesis

The P-E hypothesis conceptualizes health in old age as a direct outcome of (mis) fit between environmental press (demands of the socio-physical environment) and personal competence (the ability to cope with environmental demands) (23). Environmental press can be positive, negative, or neutral, and may range from pedestrian infrastructure affecting neighborhood walkability to neighborhood aesthetics, crime, and land-use patterns affecting physical function and mental health (24). Personal competence refers to such individual qualities as biological endowments, cognitive skills, and intelligence, which can either be high or low. In the P-E schema, a harmonious balance between press and competence would result in positive adaptation and well-being, while a misfit often leads to maladaptation and poor health outcomes.

The P-E hypothesis dominated research in environmental gerontology throughout the 1970s and 1980s, and has been influential in driving recent studies examining aging-in-place as a viable alternative to institutional care for older adults (25, 26). Other recent application of the hypothesis includes studies investigating relations between neighborhood safety and psychological health (27), and between neighborhood socio-physical environment and life satisfaction among older adults (28).

## Methods

### Research Settings

**Figure 1:**
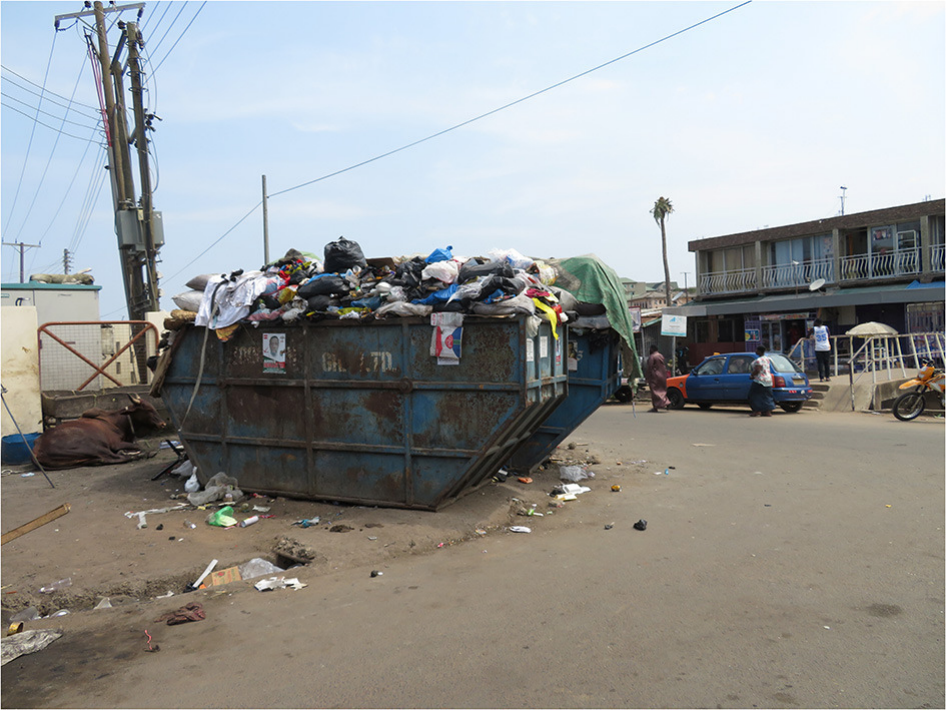
A section of Nima, Accra.

Data collection was undertaken in the Nima slum and the Adabraka-Asylum Down non-slum neighborhoods in Accra, Ghana's capital city. Historically, Nima emerged in the early 1930s as a legal but unplanned settlement, or what Majale (29) termed a “pirate settlement.” The site of the neighborhood was originally acquired as a transitional grazing ground for cattle meant for sale in the rapidly expanding city of Accra (30, 31). By the 1940s, Nima had become the centre of haphazard housing development, by mostly migrant workers seeking employment in a nearby military base to the northeast and a wealthy European neighborhood to the south of the settlement (32). Nima was not incorporated into Accra's metropolitan boundaries or subject to urban planning regulations until the 1950s, when the neighborhood was already a mature slum (31). This neighborhood is today the largest Ghanaian urban slum, with limited access roads and comparatively poor housing and sanitation infrastructure (32, 33) (Figure 1).

Nima is also highly congested and inhabited predominantly by people of the Islamic faith. In 2010, the neighborhood had a population of ~81,000, distributed across a land area of only 1.6 km^2^ (34, 35).

**Figure 2:**
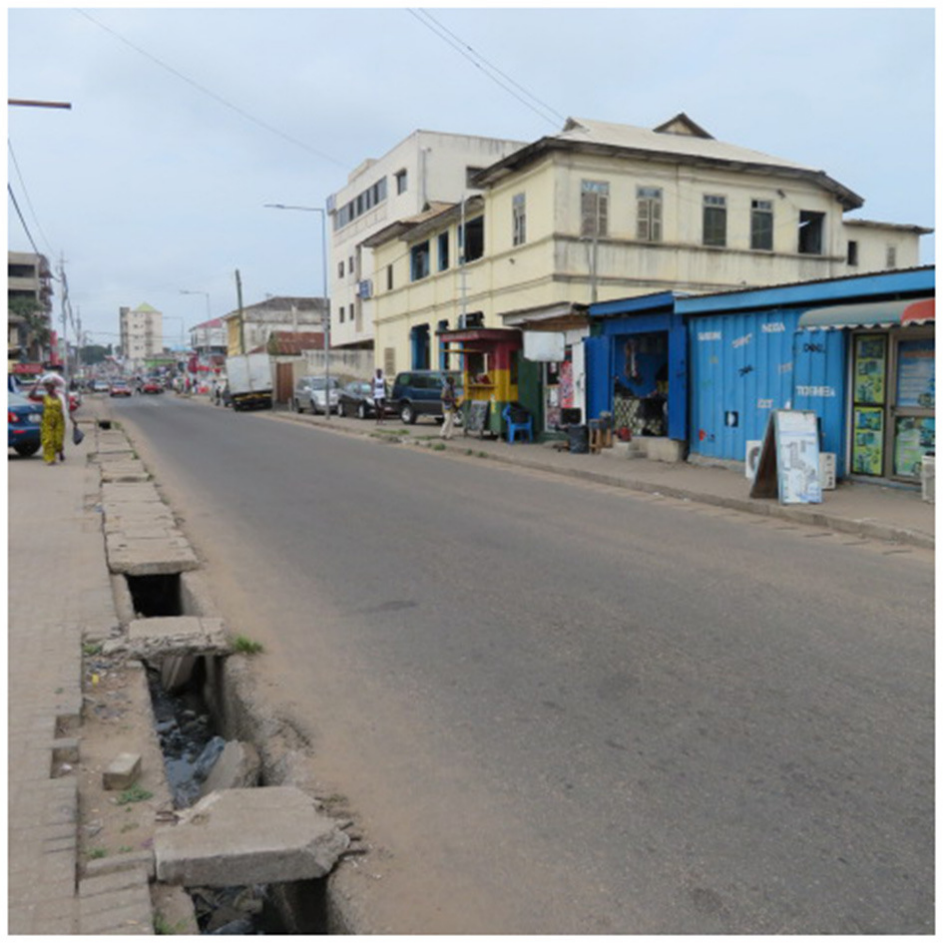
A section of Adabraka, Accra.

Established in 1910, Adabraka-Asylum Down is a planned, middle-income neighborhood, located 2km from downtown Accra (36, 37). Compared to Nima, the Adabraka-Asylum Down neighborhood has an interconnected network of streets and a functional drainage system (38), although its southern border is flood-prone due to inappropriate and often unapproved upstream land-use activities (39). The neighborhood's location near the city's central business district (CBD) also makes it a bustling hub for commercial activities (40). The socio-environmental characteristics of the slum and non-slum offered a useful contrast for a comparative analysis of barriers and facilitators to health among older adults (Figure 2).

### Design and Recruitment

An ethnographic methodology informed the research design and data collection process. Ethnography is concerned with “what people *do* as well as what they *say*” [(41), p. 552]. By immersing in the cultural worlds of others, ethnographers can gain insider perspectives on the organization of human societies. Ethnographic approaches involving qualitative interviews, focus groups, and (non) participant observations have grown increasingly popular among researchers examining neighborhood health (22, 42). Following approval from the ethics review committees of the University of Alberta and the University of Ghana, two phases of data collection were completed in the summer of 2018. The first phase involved semi-structured interviews with a purposive sample of older adults residing in the slum and non-slum neighborhoods. The individuals selected for participation were those aged 60 years or older, as officially adopted in Ghana's National Aging Policy [(43), p. 37]. Participants were also those deemed capable of providing rich information and who expressed interest in participating in the study. An open-ended interview guide informed by the neighborhood-health literature and the person-environment hypothesis guided the conduct of these interviews. The guide solicited participants' perceptions of neighborhood health barriers and facilitators, in particular the socio-environmental factors influencing their health and well-being. These interviews lasted ~1 to 1.5 h long and were conducted in the homes of participants. Each participant was given a GHC 20 gift certificate.

The second phase involved semi-structured interviews with health workers and community leaders. The health workers were physicians and public health nurses serving residents of the two neighborhoods, while the community leaders were elected local government representatives. These participants responded to questions about the living conditions of older adults in the two neighborhoods and the health challenges confronting them. The interviews with health workers were conducted at the healthcare facilities where they worked, while those with community leaders occurred in the communities. Each participant provided a written consent prior to the interviews. All interviews were conducted face-to-face, in English, audio-recorded, and transcribed verbatim by a professional transcriptionist before analysis. The first author conducted all interviews. Data saturation was reached when subsequent interviews became informationally redundant (44), after integrating responses across all three categories of participants. Fieldnotes, based on researcher reflections and observations, were recorded throughout the entire data collection period.

### Data Analysis

Aided by NVivo 12 (45), the data analysis process was thematic, simultaneously inductive and deductive, and involved using the data management functions of the software to condense the data into nodes, categories, and subsequently themes, reflecting older adults' perception of health barriers and facilitators. The hybridization of data-driven (inductive) and theory-driven (deductive) approaches to thematic analysis is an emerging paradigm in qualitative research that supports a more nuanced understanding and interpretation of research data (46).

The inductive analysis followed Green et al.'s (47) 4-steps analytical framework of data immersion, coding, creating categories, and identifying themes (p. 547). Data immersion was undertaken throughout the period of data collection, and continued thereafter through repeated reading of the interview transcripts. This iterative analytic strategy allowed for modification of the interview guide in response to emerging data gaps in the field. Through the process of immersion, we were able to develop familiarity with the data and create a codebook of emerging nodes, or groups of meaningful statements. In the second step, the transcripts were imported into NVivo 12 coded inductively using the codebook developed previously. The coding process involved sorting individual words, phrases, and paragraphs in the transcripts into nodes. The third step involved exploring relationships between nodes, sorting nodes, and merging nodes into categories that illuminated particular aspects of the research objectives. In the final step, the categories were merged into themes that reflected patterns of barriers and facilitators to health in the slum and non-slum. The formation and interpretation of the emerging themes followed a deductive process that relied on the explanatory power of the *person-environment hypothesis*.

Data triangulation, reflexive memos, and an engaged advisory committee afforded opportunities for methodological rigor (48). Data triangulation was achieved by interviewing multiple stakeholders, including older adults, community leaders, and health workers. The reflexive memos were based on documentation of how our positionalities as non-community members influenced our emerging understanding of the data. Multiple debriefing sessions with a multidisciplinary advisory committee provided additional opportunities for methodological rigor.

## Results

### Sample Characteristics

The sample consisted of 15 community-residing older adults in each neighborhood, a community leader from each, and 3 and 2 health workers from the slum and non-slum, respectively. The older adults in the sample were, on average, 70.5 years old and had been living in their current neighborhood for 48.2 years prior to the study (Table 1). These participants were predominantly male (63.3%), Christian (70%), and regular income earners (66.7%). There were a few notable differences in sample characteristics between the slum and non-slum. For example, compared to the non-slum, there were more males and Muslims in the slum sample. The higher proportion of Muslims in the slum sample reflected the neighborhood's predominantly Muslim population.

**Table 1:** Demographic characteristics of older adult participants.

**Variables**	**Total (*N* = 30)**	**Slum (*n* = 15)**	**Non-slum (*n* = 15)**
Age, mean (SD)	70.5 (7.4)	70.7 (8.0)	70.3 (7.0)
Years stayed, mean (SD)	48.2 (18.5)	47.1 (19.3)	49.3 (18.4)
**Gender**, ***n*** **(%)**			
Male	19 (63.3)	11 (73.3)	8 (53.3)
Female	11 (36.7)	4 (26.7)	7 (46.7)
**Religion**, ***n*** **(%)**			
Christian	21 (70.0)	6 (40.0)	15 (100.0)
Muslim	9 (30.0)	9 (60.0)	–
**Living arrangements**, ***n*** **(%)**			
Alone	3 (10.0)	1 (6.7)	2 (13.3)
With family	27 (90.0)	14 (93.3)	13 (86.7)
**Homeownership**, ***n*** **(%)**			
Own/family property	21 (70.0)	9 (60.0)	12 (80.0)
Tenant	9 (30.0)	6 (40.0)	3 (20.0)
**Have regular income**, ***n*** **(%)**			
Yes	20 (66.7)	7 (46.7)	13 (86.7)
No	10 (33.3)	8 (53.3)	2 (13.3)

### Health Barriers

#### Poor Built Environments

Open drains containing stagnant water and liquid waste were widespread in the slum, creating suitable breeding grounds for mosquitos, and subsequently malaria infections. Health workers in the slum identified malaria and fever as common causes of morbidity among older adults presenting at their facilities. While open drains were less common in the non-slum, its built environment presented similar kinds of health risks. A significant portion of this neighborhood lies within the floodplain of the Odawna River, where flooding resulting from unregulated upstream residential development was frequent. Perennial flooding along the river bank was a source of health and safety concerns among older non-slum dwellers. A particular health threat posed by the floods was the transmission of flood-related infectious diseases, such as cholera, malaria, and pneumonia.

It is a slum, to a large extent…(with) dirty gutters, littered streets, and open water sources for mosquitos. The refuse is everywhere…The environment is not healthy. (So)…Commonly…the usual infections, whether urinary tract infections, malaria, or pneumonias. – HWNI002 (Slum health worker).

The challenges I face here is this flood issue…The water level was seven feet in our rooms. It affected me. I was sick. I had pneumonia…because they excrete themselves in the gutter. All the water that comes here is full of human excreta. – AAP15 (Male non-slum participant).

The open drains and uneven surfaces in the slum also posed significant safety hazards to its older residents. A parallel safety hazard in the non-slum was the dilapidation of buildings along the banks of the Odawna River, where the physical impacts of the flood waters were most severe.

Recreational walking in the slum was severely impeded by its lack of streets and sidewalks. This difficulty was exacerbated by competing demands for open space from pedestrians, traders, motorists, and domestic animals within the slum's vast expanse of closely built houses and tenements. The lack of access roads and paved sidewalks impeded the free flow of traffic and prevented older adults from undertaking outdoor physical activities, such as walking and running. In contrast, the non-slum neighborhood was spatially planned and had an interconnected network of roads. Nevertheless, the streets and sidewalks of the non-slum were occupied by traders who colonized these spaces for displaying their goods, making them inaccessible for recreational walking in much the same way as the slum.

So, instead of us to have pavements to walk on, you would see that traders have taken over the pavements and we all have to jam up on the road. So, pedestrians don't have their way. We mix with [moving] vehicles and motorbikes…If I want to board a vehicle now, in fact you would see how cautious I would be. I have to make sure that I walk slowly and be dodging some vehicles and so forth. – NIP001 (Male slum participant).

We need (the) pavements. They should clear the road. Even you can see opposite the house there. They (traders) are back on the pavement. If you go to Adabraka, by the Adabraka Market, they've all built something on the sidewalk. – AAP015 (Male non-slum participant).

The fear of falling into open gutters or colliding with moving vehicles was reported to have played a role in confining large numbers of slum and non-slum older adults to their immediate home environments. This problem was especially salient for those using wheelchairs and walking aids.

#### Housing Precariousness

The majority of slum residents experienced poor ventilation due to poor housing construction methods, which relied mostly on flimsy building materials (e.g., mud bricks, overaged corrugated iron sheets, plywood, etc.). As such, most slum buildings were structurally weak and substandard, producing living conditions that were largely unsuitable for older adults. A female slum participant described her home as “a muddy structure (with) not a single concrete block” (NIP010). A health worker added that the housing situation in the slum negatively “impacts the elderly, some (of whom) get pneumonia because they are not getting proper ventilation” (HWNI003). The slum housing also provided insufficient living space, as a single bedroom unit typically accommodated as many as 10 occupants.

Although a planned neighborhood, the non-slum had an aging housing stock dating far back to the colonial era. A significant proportion of homes in this neighborhood was therefore reported to be in a state of disrepair, with essentially similar kinds of health risks as the slum – e.g., damp and moldy housing conditions, visible signs of infestations, etc.

We live in a congested house that does not provide me with much freedom. As I am telling you, I have three children with a wife – five of us – we are living in a single room and a small porch. I don't think anybody would be happy in that accommodation. It affects my health. How many beds can we have in a (single) room? To be healthy, you have to sleep well. And that is what I don't have here. We wake up very tired. – NIP002 (Male slum participant).

As for the house and my room, you know, the building is old. What I am experiencing is weak windows, weak doors. You know, in the olden days, they used wood to do the flooring. Adabraka is sitting on water. So when there is much rain, sometimes the water from underground enters through the patches and come into the room. This morning, behind there, you could see that the place is a little bit wet. – AA001 (Female non-slum participant).

For older slum dwellers, the challenges of living in overcrowded and poorly ventilated housing included sleep deprivation. In the non-slum, some participants attributed their respiratory health problems to the cold, damp, and moldy housing conditions in which they lived.

Private sector-led gentrification of the housing stock in the non-slum exacerbated the housing precariousness facing its older residents. The redevelopment and conversion of the affordable housing stock into shops, condominiums, and hostels contributed to rent hikes and growing homelessness among older adults residing in the non-slum.

Some (older adults) are even squatters. And as I'm talking to you, I have somebody who is sleeping outside who is 60 years. So it is to do with the money for the accommodation. After pension, because their (pension) money is so small and the (rent) increments have come, by the time you realize, the person is sleeping outside. – CLAA001 (Non-slum community leader).

According to the health workers, housing instability and homelessness were a pathway to health-damaging exposures, including hunger, sleep deprivation, physical insecurity, and mental health problems among older adults.

#### Unsanitary Living Conditions

In both neighborhoods, participants reported facing unsanitary conditions, including a complete absence of toilets in some homes. This problem was noticeably more severe in the slum, where the majority of residents used public toilets and practiced open defecation. Traveling to and queueing at public toilets was both time-consuming and physically grueling for older adults, especially those with impaired mobility or compromised bowel and urine control.

As there is no toilet in the house, I have to use the public toilet. But sometimes, there are long queues at the public toilet, and it is a problem if you need to use the toilet now, now, (and) now. And using the public toilet is expensive. – NIP015 (Male slum participant).

So, you see, when you walk around, all this gives us sickness, because the rubbish is everywhere, especially these plastic (bags). Some people defecate in it, then leave it anywhere. There are some places where they don't have toilets. – AAP005 (Male non-slum participant).

Study participants reported fecal contamination in both neighborhoods, due to widespread open defecation practices. They associated contact with raw sewage with a variety of health hazards, including cholera and other enteric illnesses.

A lot of houses in this community have no toilets. So, open defecation is a huge problem. So, the moment the rains come, then you start to get cholera. gastroenteritis, diarrhea, and vomiting. – HWNI001 (Slum health worker).

It always brings sickness. Even 1 day, I had to be rushed to the hospital because of this cholera outbreak. It affected me, so they had to rush me to the Adabraka Polyclinic. And I went on admission there for about 4 days before I came back. – AAP011 (Female non-slum participant).

The slum's sanitation problem was exacerbated by indiscriminate disposal of solid waste, usually into open gutters and streets. Accordingly, more slum than non-slum participants reported vulnerability to environmental afflictions arising from such practices.

People (in Nima) put trash in the gutter. We don't care about the environment at all. Somebody can carry his whole dustbin and go and put in the gutter. When you put trash inside the gutter, water accumulates there, what is the result? It would breed mosquitoes. The mosquitoes bring malaria. Sometimes, I get malaria. – NIP005 (Female slum participant).

The turbid drains and pungent ambiance were also a source of health concern to the slum participants. A participant vented: “I don"t like dirty things. So, always, I am annoyed, especially when I'm going to the (food) market.” – NIP010 (Female slum participant). Another added: “My BP (blood pressure) is not agreeing with that breeze, (and) it can give me sickness.” – NIP014 (Male slum participant).

#### Defective Public Services and Amenities

Deficiencies in access to potable water and waste collection services were significant concerns in both neighborhoods. The slum participants expressed grave concern over the environmental health risks posed by piles of uncollected refuse around their homes. They blamed the situation on poor performance of the Accra Metropolitan Assembly (AMA), the agency responsible for the city's waste collection services. While the non-slum participants were equally dissatisfied with the services of the AMA, they were largely able to address the service deficiencies by hiring private pay-for-service operators.

We sweep our houses and the refuse is supposed to be sent to a dumping site. But our dumping inside here, there is no time you will get there and see an empty container. Anytime you go there, it is full. Because when they bring the container, those around there see it first, and they rush there. – NIP009 (Male slum participant).

Years back, it's the AMA that cleans the drainage (of) the rubbish. Now, they are forcing us to clear the rubbish from the drainage. Why do we pay property rate? We pay property rate for these services. We pay property rate. So something has got to be done. – AAP015 (Male non-slum participant).

While private pay-for-service waste management operations contributed to a more appealing outdoors in the non-slum, such arrangements were limited in much of the slum. Consequently, the slum participants reported more rodent infestation of their homes and surroundings.

Both neighborhoods also experienced acute water shortages, despite the presence of water supply infrastructure, including community pipes. As such, residents had to trek long distances in search of water, a task many older adults were unable to perform.

I'm very, very worried about the water. The water is a problem, because nowadays I can't carry (water). My neck, I have a problem. My chest too has a problem. I can say my spinal cord, something like that. So, to carry water is hard. So as for water, it's difficult for me. Water problem is difficult for me. – NIP014 (Male slum participant).

Most of the time, they put the water off, and you have to carry a bucket, go to other places before you get small water and come, and then use it to do what you have to do, which at times pains all of us. – AAP013 (Female non-slum participant).

Older adults who lacked family support had to either pay as high as 40 pesewas/liter for water from private suppliers or forego such necessities as washing, cleaning, and bathing.

#### Social Incivilities

Crime and noise pollution were prevalent in both the slum and non-slum. Located just 5 km apart, the two neighborhoods reportedly had similar crime rates. The slum was perceived as harboring some of the Ghanaian police most wanted criminals. Although arm robbery and drug-related crimes were also common in the non-slum, most of these crimes were said to be spillovers from the slum.

Thieves, arm robbers, most of them are from Nima. When we came here in those days, oh, anytime you hear of thieves (it was in Nima). Even thieves (would) move from Nima to other places to steal and come. – NIP009 (Male slum participant).

The only problem, I will say, is due to those people from Nima and other places who have been patrolling in the nights collecting people's phones and their money. There was a time even they snatched my phone. – AAP014 (Male non-slum participant).

Older adults adapted to neighborhood crime by restricting their movement to daytime and within short distances from their homes. The slum's reputation as an abode for criminals also affected access to employment and public services for its residents. The residents were reportedly blacklisted by employers and service providers, and as such could not, for example, “go to hospital” and expect proper care without having to “forge someone's house number” in another neighborhood. – NIP004 (Female slum participant).

Although the city has bylaws regulating noisemaking, such regulations were generally unenforced. The resulting human and vehicular noise pollution served as a source of discomfort to older adults in both neighborhoods.

There's some house here. Every day, the younger people around that area have an (entertainment) program. Sometimes, those smokers, they have some program, and they would come and be playing music at high volume in the night. They don't sleep – midnight, the area people can't sleep. They complain bitterly. – CLNI001 (Slum community leader).

They make noise, especially the churches. The small, small churches, they make noise. And if they are making their outdooring and their weddings, they disturb. As for me, I want a quiet place. – AAP004 (Female non-slum participant).

For older adults, noise pollution was emotionally and psychologically unsettling, as it disrupted their sleep and mental concentration. A slum participant identified “fright,” “unusual heartbeat,” and “sleep problems” as some of the health effects of excessive noise in her neighborhood. – NIP007 (Female slum participant). Another felt that his hypertension was exacerbated by the noise pollution and sleep disruption he experienced in the slum: “That's why I have hypertension, because in the night, sometimes you hear some shout. You'll be frightened and then wake up.” – NIP011 (Male slum participant). Several others mentioned the impact of noise pollution on their mental health: “You cannot have peace of mind and you cannot have good sleep. If I don't have enough rest, you see that in the day I am disturbed.” – NIP001 (Male slum participant).

### Health Facilitators

#### Affordable Housing

The slum provided shelter to a large number of older adults who, for financial reasons, could not rent mainstream housing elsewhere at the prevailing market rate. Accordingly, the slum participants described their substandard shelter as a relief from exorbitant rentals and possible homelessness. They described the cost of renting in the slum to be comparatively lower and more affordable.

I like Nima, because this is where I can get cheaper accommodation to live. So, I prefer here because there is nowhere I can get a room to rent at the cost that I am paying here. Accommodation is (somewhat) cheaper in Nima. The landlord doesn't even care if you don't pay. – NIP002 (Male slum participant).

In some instances, the magnanimity of landlords exempted indigent older adults from rent payments. According to participants, it was common practice for older slum dwellers to reside in rent-free housing.

If the person is in the house for a long time before he gets old, (and) can't do anything and he has no any children around him, some of the landlords used to lift that (rent) burden on them. – CLNI001 (Slum community leader).

Others were allowed free overnight stays in various Mosques across the slum. The availability of affordable and rent-free accommodation in the slum provided a safety net against elderly homelessness and its related health hazards, including physical and mental stress.

#### Neighborhood Appeal

As a spatially planned and well-demarcated neighborhood, the non-slum outdoors were aesthetically more appealing than those of most low and middle-income neighborhoods in the city. Participants, therefore, described the non-slum as “a very nice place” and one to “feel proud of staying in.” – AAP014 (Male non-slum participant). The neighborhood enjoyed an additional reputation as a political enclave, having previously hosted some of the most influential figures in Ghanaian politics, including a former president, a sitting traditional ruler, and a multitude of current and former government ministers: “Our former president, J.J Rawlings, stayed here before. Quarshigah stayed here.” – AAP010 (Male non-slum participant). The neighborhood's aesthetic appeal and rich political history together invoked a sense of pride and prestige, which were perceived to have ultimately benefitted the mental and psychosocial well-being of its older residents. It was also a major transportation hub, with transport networks reaching almost all parts of the city. A participant noted: “(If) I want to go somewhere, it's easy for me to get car.” – AAP004 (Female non-slum participant).

#### Social Supports and Neighborliness

In both neighborhoods, participants expressed satisfaction with the cordiality and social supports accorded by family, friends, religious bodies, and neighbors. They enjoyed financial and material support, as well as assistance with undertaking mundane chores.

For about 5 years now, I have not been to the Mosque. But they come to visit me, praying for me. We chat sometimes. When it comes to fasting time (Sallah festivities), they bring me food. Somebody would just come, “Oh take this 5 cedis. Take 10 cedis. Oh, I have bought you cloth (dress).” They support me. – NIP003 (Female slum participant).

There is a neighbour in the house, a tenant. She has taken me like her mother. They help me with everything…even cooking. If I say I can't cook, they will come and cook for me. – AA006 (Female non-slum participant).

Although social supports for older adults existed in both neighborhoods, the supports received in the slum were said to be superior and much more institutionalized, in accordance with Islamic teachings. For example, in addition to receiving financial and material supports from neighbors, some older slum dwellers reportedly also enjoyed rent-free housing, all of which went into enhancing their physical, emotional, and psychological wellness.

## Discussion

Guided by the P-E hypothesis, this study identified factors influencing the health and well-being of older adults residing in two contrasting residential neighborhoods in Accra, Ghana. The reported susceptibility of older slum and non-slum dwellers to infectious and parasitic diseases is consistent with findings from previous studies with older urban populations in the region (49, 50). However, in low-income urban communities such as slums, older adults' vulnerability to such inflictions is likely to be higher, given what may appear to be a mismatch between their personal competences and the environmental press presented by the residential settings in which they live (23). The environmental press identified in the present study (e.g., clogged open drains and piles of uncollected refuse in the slum and floodwaters in the non-slum) supports this assertion and may have contributed to the disease burden of older adults whose health was possibly already under threat from a plethora of non-communicable illnesses (51). Yet, health programs specifically targeting the unique healthcare needs of older adults are almost nonexistent in the Ghanaian context (52). A community-based primary care model integrating both treatment and preventive interventions would help to address the double-burden of disease confronting older Ghanaians residing in low-income settings. The National Health Insurance Scheme (NHIS) could, for example, be better positioned to deliver an integrated model of care for low-income older adults, including those residing in the studied neighborhoods. Social policies targeting improvements in the living conditions of older adults are also warranted, given the observed relationship between poor housing and ill-health.

Mobility and recreational walking among older adults in the slum were curtailed by the neighborhood's spatial disorganization, particularly the open drains and limited streets and sidewalks that characterized much of its built environment. The walkability of the non-slum was similarly affected by vehicular traffic and encroaching street traders who seized much of the available space for their business activities. This environmental press reflects a larger systemic problem in the urban architecture of SSA, where vehicular-dependency predominates and spaces for walking remain limited (53). The near absence of dedicated spaces for walking, running, and biking appears to contribute to physical inactivity, particularly among older adults whose fear of the outdoors may partly be responsible for the rising levels of obesity, diabetes, and cardiovascular diseases in African cities (54). As the population of urban older adults is projected to rapidly increase, neighborhoods must be (re) designed to ensure they are safe for physical activities.

Excessive noise pollution was a general issue of public concern in the city (55). For older adults, this upheaval was intolerable, as it presented a common environmental press affecting their health and well-being. Our observations conformed with an emerging scientific consensus suggesting a general decline in noise tolerance with increasing age, a physiological change widely associated with hypersensitivity of the aging brain (56, 57). Excessive neighborhood noise can cause sleep disruption and mental health problems for older adults (58), who, owing to their advance age, may require a greater amount of uninterrupted rest. The participants' experiences of anxiety and irritation arising from neighborhood noise reflected this general pattern. A similar P-E misfit was reported in relation to older adults' experience with neighborhood crime, whether actual or perceived. Consistent with earlier observations in the United States (16, 59), this study found fear of crime to play a role in restricting free movement of older adults.

Older adults in both neighborhoods reported experiencing housing precariousness. In the slum, most residents resided in overcrowded, poorly ventilated, and unsanitary housing conditions with limited access to potable water and safe sanitation, which, according to participants, increased their risk of respiratory and sleep problems. The gentrification of the overaged housing stock in the non-slum contributed to homelessness among older adults in this neighborhood. The housing experiences of older adults in both neighborhoods resonate with those of their counterparts residing elsewhere in the sub-Saharan African region. In Kenya, for example, overcrowding in slums remains a key challenge to the health and well-being of older adults, despite recent attempts at upgrading (60). Although private sector-led slum upgrading and gentrification of decaying neighborhoods are often well-intended, human rights activists have remained skeptical, particularly in the face of mounting evidence suggesting displacement of poorer residents in areas where these initiatives have been implemented (61). This discourse suggests a need for state-led interventions to improve the health and well-being of low-income older adults. Large-scale public sector investment in social housing is currently underway in Brazil and India (62), and Ghana could learn valuable lessons from such ambitious initiatives as it attempts to improve the residential conditions of low-income older adults.

The experience of aging in the two neighborhoods was not overwhelmingly negative. In fact, several attributes of the two neighborhoods mitigated, to some extent, the harmful effects of the identified health threats. The non-slum residents benefitted psychosocially from their neighborhood's aesthetic appeal and prestige as a political enclave for famous Ghanaian politicians and statesmen. Similarly, slums may be reimagined as ambiguous places offering both “hope” and “despair” (63). Consistent with the literature (64, 65), the slum neighborhood indeed possessed certain therapeutic qualities – e.g., affordable housing, social supports, and a strong sense of community belonging. In particular, a culture of gift-giving, enshrined in Islamic ethos (66), provided a safety net against poverty and destitution among older slum dwellers.

## Summary and Conclusion

Our qualitative inquiry identified several barriers to health among older slum and non-slum residents. Open drainage systems in the slum and unregulated upstream residential developments in the non-slum were associated with infectious diseases (e.g., malaria, pneumonia, etc.), fear of physical harm, and physical inactivity among older adults. Substandard housing in the slum and an aging housing stock in the non-slum posed similar health threats to older adults. In particular, insufficient living space and poor ventilation in the slum were associated with incidences of pneumonia among older residents, while the damp and moldy conditions of the aging housing stock in the non-slum were widely seen as a risk factor for this disease. Although to varying degrees, sanitation conditions in both neighborhoods were summarily poor and inimical to the health and well-being of older adults. For example, toilet facilities and waste disposal systems were reportedly inadequate and inappropriate in both the slum and non-slum, serving as risk factors for cholera and other infectious diseases among older residents. Social incivilities such as crime and noise pollution were reported in both neighborhoods as significant sources of physical and psychological discomfort to older residents, including experiences of sleep problems and lack of concentration. The health impacts of these environmental conditions were, however, mitigated by several health-enhancing conditions, including affordable housing and generous social support systems in the slum and appealing outdoors in the non-slum. Nonetheless, public sector interventions are needed to remediate the aforementioned health risks.

In conclusion, despite distinct spatial and socioeconomic characteristics, we found similar patterns of health risks in the two contrasting neighborhoods. However, the factors underlying these risks were, in many instances, specific to each community. A more critical exploration of causal relationships points to structural determinants of health barriers, which then manifest differently at the neighborhood level as community-specific risk factors for poor health. For example, urban poverty and a neoliberal development paradigm undermining the needs of older adults are largely responsible for their poor living conditions across the city. Given these broader observations, enhancing the health and well-being of slum and non-slum older adults would, among others, require improvements to their current housing and neighborhood conditions, including their access to municipal services, crime-free outdoors, and suitable sanitation infrastructure. It is also critical, in view of the findings, to re-examine the slum and non-slum dichotomy in Ghanaian settlement classifications. While the two neighborhoods aligned with official definitions of slum and non-slum, in terms of whether they are planned or unplanned, the lived experiences of the participants demonstrated more convergence than divergence. We therefore suggest that interventions to address health vulnerabilities among older adults be based on assessment of actual need rather than settlement classifications.

## Study Limitations

The study sample was small in size, purposive, and statistically unrepresentative of the population of older adults residing in the slum and non-slum. This limitation potentially affects the generalizability of the findings. Future research relying on statistical sampling techniques and collection of large quantitative data would help extend public health understanding of variations in health risks among slum and non-slum neighborhoods. The generalizability of our findings is further curtailed by our limited focus on two neighborhoods. Future comparative studies exploring the health of older adults could be much broader in geographical scope, covering multiple slum and non-slum settlements. Furthermore, as a qualitative study, we were unable to establish correlations between neighborhood environmental factors and the health conditions reported by participants. As such, our claims of causal relationships relied exclusively on participant narratives. Epidemiological studies are thus needed to further public health knowledge of associations between neighborhood conditions and health risks pertaining to older adults in SSA. Nonetheless, our study demonstrates that older adults, regardless of neighborhood location, face similar environmental barriers to health.

## Data Availability Statement

This paper utilized original data collected by the authors. Inquiries can be directed to the corresponding author.

## Ethics Statement

The University of Alberta Research Ethics Board and University of Ghana Research Ethics Committee reviewed and approved the study protocol. All participants provided informed written consent to participate in the interviews.

## Author Contributions

All authors contributed to the conceptualization, investigation, writing, review, and editing of the manuscript. All authors approved the submitted version.

## Conflict of Interest

The authors declare that the research was conducted in the absence of any commercial or financial relationships that could be construed as a potential conflict of interest.
